# Combining computed tomography features and histogram analysis facilitates differentiation between solitary pulmonary invasive mucinous and non-mucinous adenocarcinomas

**DOI:** 10.3389/fradi.2026.1793604

**Published:** 2026-04-14

**Authors:** Duiming Yang, Hao Hu, Zhimei Li, Shuilan Zhang, Jing Ai, Handan Zhang

**Affiliations:** 1 Department of Medical Imaging, The Second People's Hospital of Baoshan City, Baoshan, China; 2 Medical Imaging Center, The First Hospital of Kunming, Kunming, China; 3 Kunming Medical University, Kunming, China

**Keywords:** computed tomography, gray histogram, lung, mucinous adenocarcinoma, precision medicine

## Abstract

**Objective:**

To assess the value of combining computed tomography (CT) morphological and histogram features for the differentiation of solitary pulmonary invasive mucinous adenocarcinoma (PIMA) from pulmonary invasive non-mucinous adenocarcinoma (PINMA).

**Methods:**

This retrospective study analyzed the CT images and clinical data of 58 and 105 patients with PIMA and PINMA, respectively. CT histogram features were extracted after delineating regions of interest using 3D Slicer software. CT morphological and histogram features were compared between the PIMA and PINMA groups, and those that differed significantly were included in multivariate logistic regression models. The independent predictive factors identified were used to create CT morphological, CT histogram-based, and combined prediction models. The best-performing model was visualized and evaluated by constructing a nomogram.

**Results:**

The CT morphological prediction model included nodule type, vacuole sign, and tumor location as factors predictive of PIMA and had an area under the curve of 0.754. The CT histogram-based prediction model included kurtosis and the 90th percentile as factors predictive of PIMA and had an area under the curve of 0.820. The combined prediction model, which included tumor location, vacuole sign, kurtosis, and the 90th percentile, had an area under the curve of 0.845, suggesting greater diagnostic accuracy than the separate models. The combined prediction model also exhibited good calibration and high clinical applicability.

**Conclusion:**

Integrating CT morphological features and histogram analysis improves the accuracy of differentiating PIMA from PINMA. The nomogram provides a practical and effective tool for the non-invasive diagnosis of lung cancer subtypes.

## 1 Introduction

Lung cancer remains the leading cause of cancer-related mortality worldwide (1), with adenocarcinoma the most prevalent histological subtype. Pulmonary invasive non-mucinous adenocarcinoma (PINMA) is the predominant form of lung adenocarcinoma, while pulmonary mucinous adenocarcinoma (PIMA) is a relatively rare subtype, accounting for 2%–5% of all cases (2). Epidermal growth factor receptor mutations, the most common oncogenic driver in PINMA, are comparatively infrequent in PIMA. This disparity directly contributes to the marked differences in therapeutic responses to epidermal growth factor receptor-targeted agents between the two subtypes (3–5). In addition, the significantly higher rates of recurrence and spread through air spaces observed in PIMA compared to PINMA (6) indicates substantial differences in treatment response and biological behavior. Therefore, accurately differentiating between PIMA and PINMA is critical to implement optimal individualized treatment strategies.

The use of biopsy to distinguish PIMA from PINMA is limited by the availability of biopsy specimens and tumor heterogeneity, and few studies have investigated the preoperative non-invasive diagnosis of PIMA. Conventional computed tomography (CT) provides abundant information but has inherent limitations in distinguishing nodular PIMA from PINMA; the feasibility of this method remains controversial (7–9). Radiomics has shown substantial progress in disease diagnosis (10, 11); however, the complexity of model construction has hindered its widespread clinical implementation.

This study examined the potential use of CT histogram analysis to differentiate between PIMA and PINMA. This quantitative imaging technique characterizes lesion heterogeneity by analyzing the distribution of gray-level values, thereby extracting features that are difficult to capture using conventional CT. Histogram analysis places greater emphasis on the quantitative assessment of the intra-tumoral microenvironment than conventional CT and is easier to implement than radiomics-based methods. This approach has been successfully applied to the evaluation of pulmonary nodules, hepatocellular carcinoma, and pancreatic tumors, among other diseases (12–16). However, systematic studies evaluating its ability to differentiate between PIMA and PINMA are lacking. Therefore, the present study aimed to quantitatively characterize the imaging features of PIMA and PINMA using CT histogram analysis and to construct a predictive model by integrating CT histogram features with conventional CT morphological features to validate its effectiveness for preoperative diagnosis. Ultimately, this study seeks to provide new insights into individualized treatment strategies for lung cancer and promote the development of non-invasive diagnostic approaches.

## 2 Materials and methods

### 2.1 Patients

The clinical and imaging data of 163 patients who underwent surgical resection at the Second People's Hospital of Baoshan City, Yunnan Province, China between January 2019 and June 2024 and were pathologically confirmed to have lung adenocarcinoma were retrospectively analyzed.

The inclusion criteria were as follows: (i) surgical resection and pathological diagnosis, in accordance with the 5th edition of the 2021 World Health Organization Classification of Thoracic Tumors (17), of PIMA or mixed mucinous–non-mucinous adenocarcinoma with the mucinous component accounting for >50% (PIMA group), or of invasive lung adenocarcinoma lacking any mucinous adenocarcinoma component in tumor tissues (PINMA group); and (ii) preoperative chest CT performed in the month before surgery, with CT images demonstrating a solitary pulmonary nodule or mass.

The exclusion criteria were as follows: (i) mucinous adenocarcinoma secondary to primary tumors at other sites, concomitant active pneumonia, pulmonary tuberculosis, or other systemic malignancies; (ii) poor CT image quality that affected interpretation; (iii) incomplete clinical data; and (iv) antitumor treatment, such as radiotherapy or chemotherapy, administered prior to surgery.

Ultimately, 163 patients met the eligibility criteria and were included in the analysis, 58 in the PIMA group and 105 in the PINMA group.

### 2.2 CT image acquisition

All CT images were acquired in our department using a 64-slice CT scanner (Optima CT680 Expert, GE HealthCare, Chicago, IL, USA) or 256-slice CT scanner (Revolution CT, GE HealthCare). The scanning range extended from the lung apices to the level of the costophrenic angles; whole-lung scans were performed at end inspiration with breath-holding. The scanning parameters are listed in Table 1.

**Table 1:** Scanning parameters of CT.

Parameters	Unit	GE	GE
Scanner model	–	OPtima CT680	Revolution
Tube voltage	kV	120	120
Tube current	mA	100–500	120–350
Matrix	–	512 × 512	512 × 512
Slice thickness	mm	1.25	1.25
Reconstruction kernel		standard	standard

GE, General Electric.

### 2.3 Image preprocessing and segmentation

Two radiologists with five and eight years of experience in thoracic imaging, who were blinded to the pathological results, independently evaluated the CT morphological features, and discrepancies were resolved by consensus.

**Figure 1:**
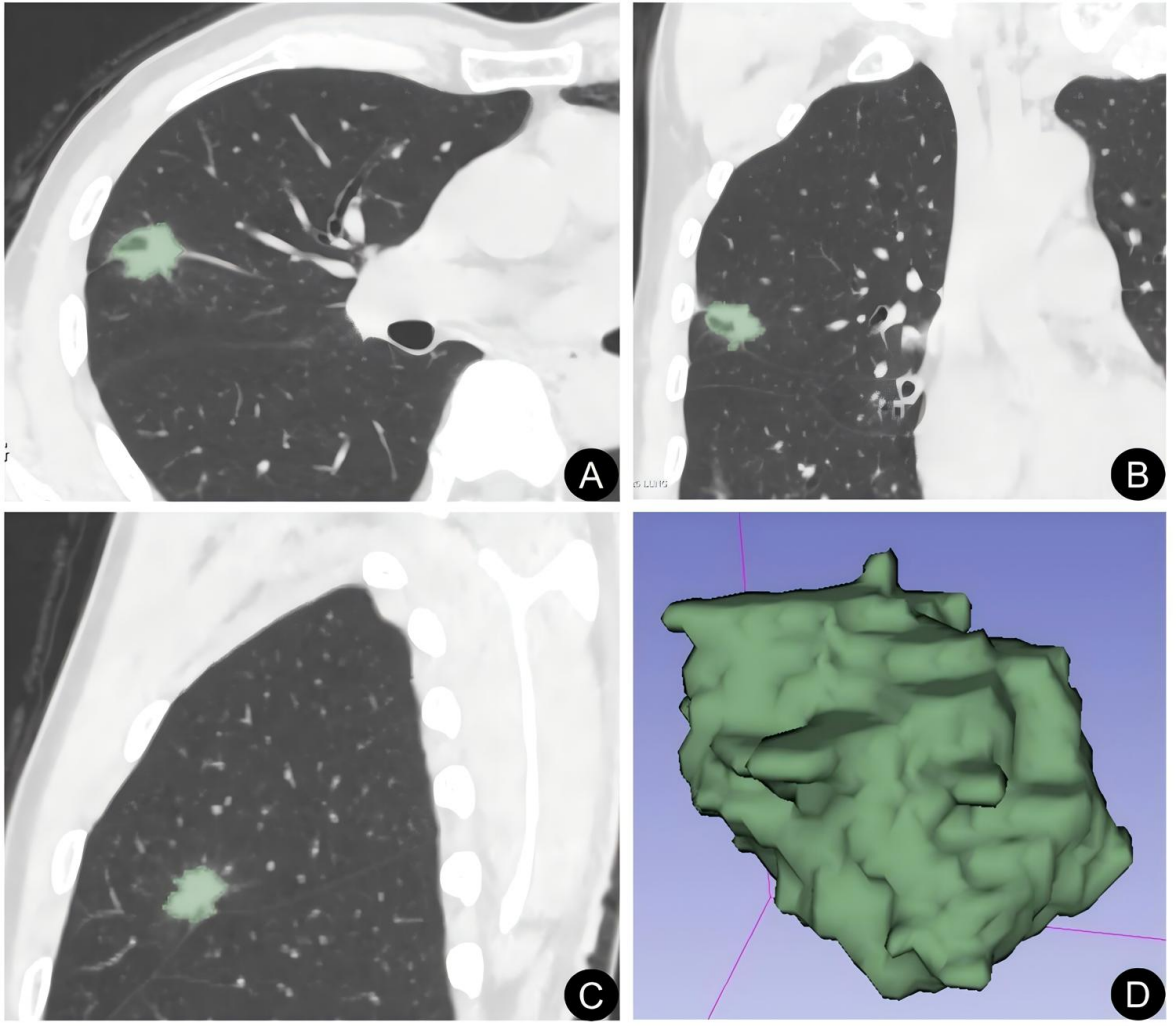
Schematic illustration of image segmentation. **(A)** axial view; **(B)** coronal view; **(C)** sagittal view; **(D)** three-dimensional visualization of the region of interest (ROI).

For the regions of interest (ROIs) segmentation, non-contrast-enhanced CT images were imported into 3D Slicer software (version 4.10.2), and the ROIs were manually delineated slice-by-slice along the lesion margins according to a standardized segmentation protocol (Figure 1). To assess inter-observer reproducibility, CT images from 30 randomly selected patients were independently segmented by the two radiologists. Histogram features were extracted from the two sets of ROIs, and the intraclass correlation coefficient (ICC) was calculated to evaluate inter-observer agreement. Features with ICC values greater than 0.75 were considered to have good reproducibility and were included in the subsequent analysis. One radiologist then performed ROI delineation for the remaining cases, and these ROIs were used for the final feature extraction. The extracted histogram parameters included variance, skewness, kurtosis, entropy, uniformity, the 10th and 90th percentiles, and the mean, maximum, and minimum CT values.

### 2.4 Statistical analysis

Statistical analyses and graphical visualizations were performed using SPSS software version 26.0 (IBM Corp., Armonk, NY, USA) and R software version 4.2.1 (The R Foundation for Statistical Computing, Vienna, Austria). Normally distributed continuous variables are expressed as mean ± standard deviation and were compared using the independent-samples t-test. Non-normally distributed continuous variables are presented as median (interquartile range) and were compared using the Mann–Whitney U test. Categorical variables are expressed as counts (percentages) and were compared using the chi-squared test. A two-sided *P* value < 0.05 was considered to indicate statistical significance. CT morphological features and CT histogram parameters that showed statistical significance in the univariate analyses were separately entered into multivariate logistic regression models to construct CT morphological and histogram-based prediction models. Before multivariate logistic regression analysis, statistically significant CT histogram parameters were assessed for multicollinearity. Redundant variables with strong collinearity were excluded based on their univariate performance, and the remaining variables were re-evaluated. Variables with variance inflation factor (VIF) values < 5 were included in the final multivariate logistic regression model. Additionally, both types of variables were simultaneously incorporated into a logistic regression model to establish a combined prediction model. Receiver operating characteristic curves were generated to determine the area under the curve (AUC), sensitivity, and specificity and evaluate the discriminatory performance of the models. Calibration curves were applied to assess the goodness of fit and decision curve analysis was performed to evaluate the clinical utility of the models.

## 3 Results

### 3.1 Comparison of clinical and CT morphological features between the two groups

Significant differences were observed between the PIMA and PINMA groups in terms of nodule type, consolidation-to-tumor ratio, bronchial morphological changes (including bronchial distortion, truncation, and stenosis), vascular abnormalities (including vascular convergence and tumor microvascular signs), vacuole sign, and tumor location (*P* < 0.05). No significant differences between the groups were identified in the tumor long-axis diameter, tumor–lung interface, pleural indentation sign, lobulation sign, spiculation sign, sex, age, or smoking history (Table 2).

**Table 2:** Comparison of clinical-CT features between PIMA and PINMA groups.

Variables	Unit	PIMA	PINMA	Test statistic	*P value*
		(*n* = 58)	(*n* = 105)	(*χ*^2^ or Z)	
Sex					
Male	–	26 (44.8)	47 (44.8)	0.000	0.994
Female	–	32 (55.2)	58 (55.2)		
Smoking history					
Yes	–	16 (27.6)	30 (28.6)	0.018	0.894
No	–	42 (72.4)	75 (71.4)		
Age	years	58.5 (50.0, 67.0)	60.0 (50.0, 67.0)	−0.593	0.553
Tumor long-axis diameter	cm	2.0 (1.4, 3.3)	1.8 (1.4, 2.4)	−1.328	0.184
Nodule type					
Solid	–	48 (82.8)	51 (48.6)	18.311	<0.001
Mixed ground-glass	–	10 (17.2)	54 (51.4)		
CTR	%				
≥50	–	55 (94.8)	80 (76.2)	9.122	0.003
<50	–	3 (5.2)	25 (23.8)		
Tumor-lung interface					
Well-defined	–	51 (87.9)	79 (75.2)	3.728	0.054
Ill-defined	–	7 (12.1)	26 (24.8)		
Bronchial morphological changes	–	19 (32.8)	17 (16.2)	5.960	0.015
Vascular abnormalities	–	15 (25.9)	67 (63.8)	25.521	<0.001
Vacuole sign	–	21 (36.2)	14 (13.3)	11.593	0.001
Pleural indentation sign	–	22 (37.9)	55 (52.4)	3.130	0.077
Lobulation sign	–	56 (96.6)	92 (87.6)	3.568	0.059
Spiculation sign	–	25 (43.1)	38 (36.2)	0.753	0.386
Tumor location					
Upper and middle lobes	–	23 (39.7)	69 (65.7)	10.320	0.001
Lower lobes	–	35(60.3)	36(34.3)		

Data are presented as *n*–(%) or median (interquartile range). CTR, consolidation-to-tumor ratio.

**Figure 2:**
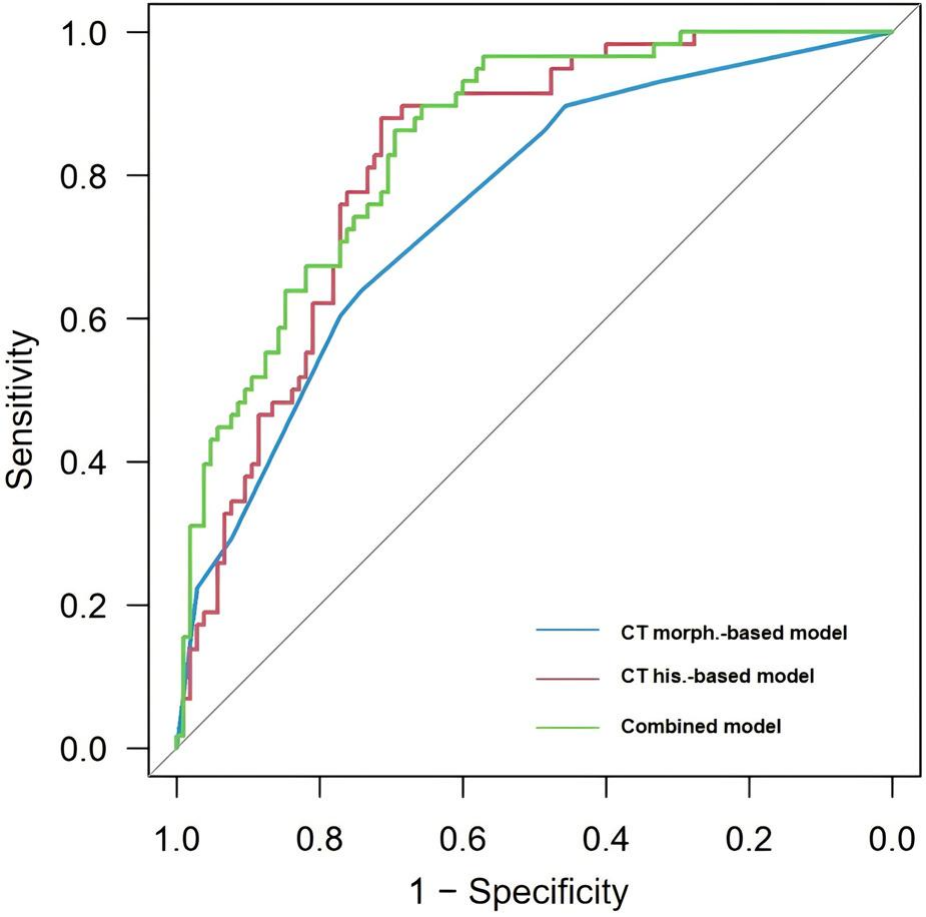
The ROC curves of models. ROC, receiver operating characteristic.

CT features that demonstrated statistical significance were subjected to logistic regression analysis, which identified nodule type, vacuole sign, and tumor location as independent predictors of PIMA (Table 3). These factors were used to establish a CT morphological prediction model, yielding an AUC of 0.754, sensitivity of 0.638, and specificity of 0.743 (Table 4 and Figure 2).

**Table 3:** Results of multivariate logistic regression analysis with CT feature and histogram features.

Variables	*P*	*OR*	95% *CI*
CT morphological features			
Nodule type	<0.001	4.511	1.998–10.184
Vacuole sign	0.008	3.118	1.343–7.240
Tumor location	0.026	2.257	1.102–4.625
Histogram features			
Kurtosis	0.040	1.005	1.000–1.011
90Percentile	<0.001	1.321	1.133–1.540
**CT morphological + histogram features**			
Tumor location	0.046	0.444	0.200–0.985
Vacuole sign	0.007	3.503	1.339–8.772
Kurtosis	<0.001	1.336	1.139–1.566
90Percentile	0.040	1.224	1.011–1.365

OR, odds ratio; CI, confidence interval. The combined model includes CT morphological and histogram features.

**Table 4:** Comparison of diagnostic efficiency of different models.

Model	*AUC*	Sensitivity (%)	Specificity (%)	95% *CI*
CT feature-based model	0.754	0.638	0.743	0.644–0.831
Histogram-based model	0.820	0.879	0.714	0.756–0.884
Combined model	0.845	0.828	0.705	0.786–0.904

AUC, area under the receiver operating characteristic curve; CI, confidence interval.

### 3.2 Comparison of CT histogram features between the two groups

The inter-observer reproducibility analysis demonstrated that all extracted histogram features showed good agreement between the two radiologists, with all ICC values exceeding 0.75. The 90th and 10th percentiles, kurtosis, maximum, mean, and uniformity were significantly higher in the PIMA group than in the PINMA group, whereas skewness and entropy were significantly lower in the PIMA group than in the PINMA group (*P* < 0.05). No significant differences were observed between the two groups in terms of the minimum and variance (*P* > 0.05) (Table 5). Among the statistically significant histogram features, several variables showed multicollinearity. After exclusion of redundant variables, those with variance inflation factor (VIF) values < 5 were included in the multivariable logistic regression analysis. Multivariable logistic regression analysis identified kurtosis and the 90th percentile as independent predictors of PIMA (Table 3). Histogram features that showed statistical significance were subjected to multivariate logistic regression analysis, which identified kurtosis and the 90th percentile as independent predictors of PIMA (Table 3). These factors were used to establish a CT histogram-based prediction model, yielding an AUC of 0.820, sensitivity of 0.879, and specificity of 0.714 (Table 4 and Figure 2).

**Table 5:** Comparison of CT histogram features between PIMA and PINMA groups.

Variables	Unit	PIMA	PINMA	Test statistic	*P*
		(*n* = 58)	(*n* = 105)	(χ^2^ or Z))	
10 Percentile	HU	−379.85 (−505.85, −270.85)	−552.60 (−648.55, −386.45)	−4.511	<0.001
90 Percentile	HU	49.00 (26.65, 59.00)	−7.00 (−135.85, 37.00)	−5.451	<0.001
Kurtosis	–	5.11 (3.44, 8.00)	1.71 (1.32, 3.28)	−7.323	<0.001
Maximum	HU	80.50 (57.00, 102.50)	62.00 (4.50, 97.50)	−2.376	0.017
Mean	HU	−99.13 (−181.02, −43.68)	−259.47 (−398.16, −107.44)	−4.579	<0.001
Minimum	HU	−776.00 (−854.25, −681.00)	−781.00 (−823.00, −725.00)	−0.309	0.758
Skewness	–	−1.49 (−2.19, −0.88)	−0.47 (−1.39, −0.13)	−4.832	<0.001
Variance	HU^2^	32,027.72 (21,440.02, 41,809.47)	34,043.80 (21,033.51, 43,954.65)	−0.409	0.683
Uniformity	–	0.10 (0.06, 0.16)	0.06 (0.04, 0.10)	−3.941	<0.001
Entropy	—	3.98 (3.44, 4.42)	4.51 (3.91, 4.74)	−3.941	<0.001

Values represent Hounsfield units (HU).

### 3.3 Development of the combined prediction model

**Figure 3:**
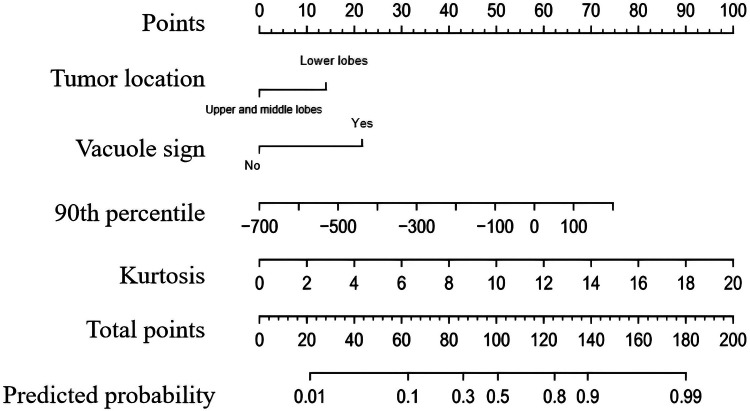
The nomogram of the combined model.

**Figure 4:**
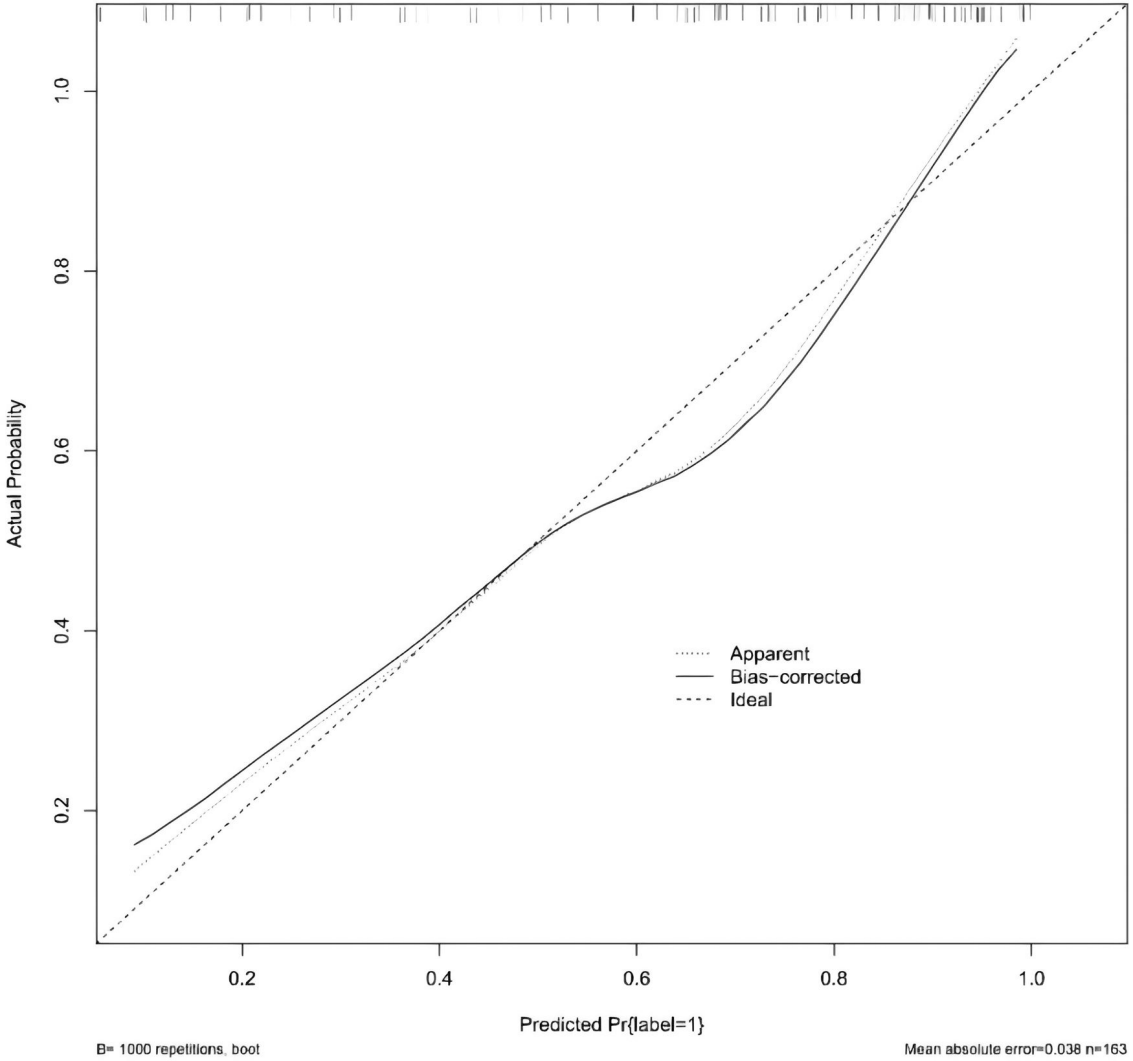
The calibration curve of the combined model. The thick dashed line represents the observed outcomes, the thin dashed line represents the model-predicted probabilities, and the solid line represents the bias-corrected performance of the nomogram obtained by bootstrapping (1,000 resamples).

**Figure 5:**
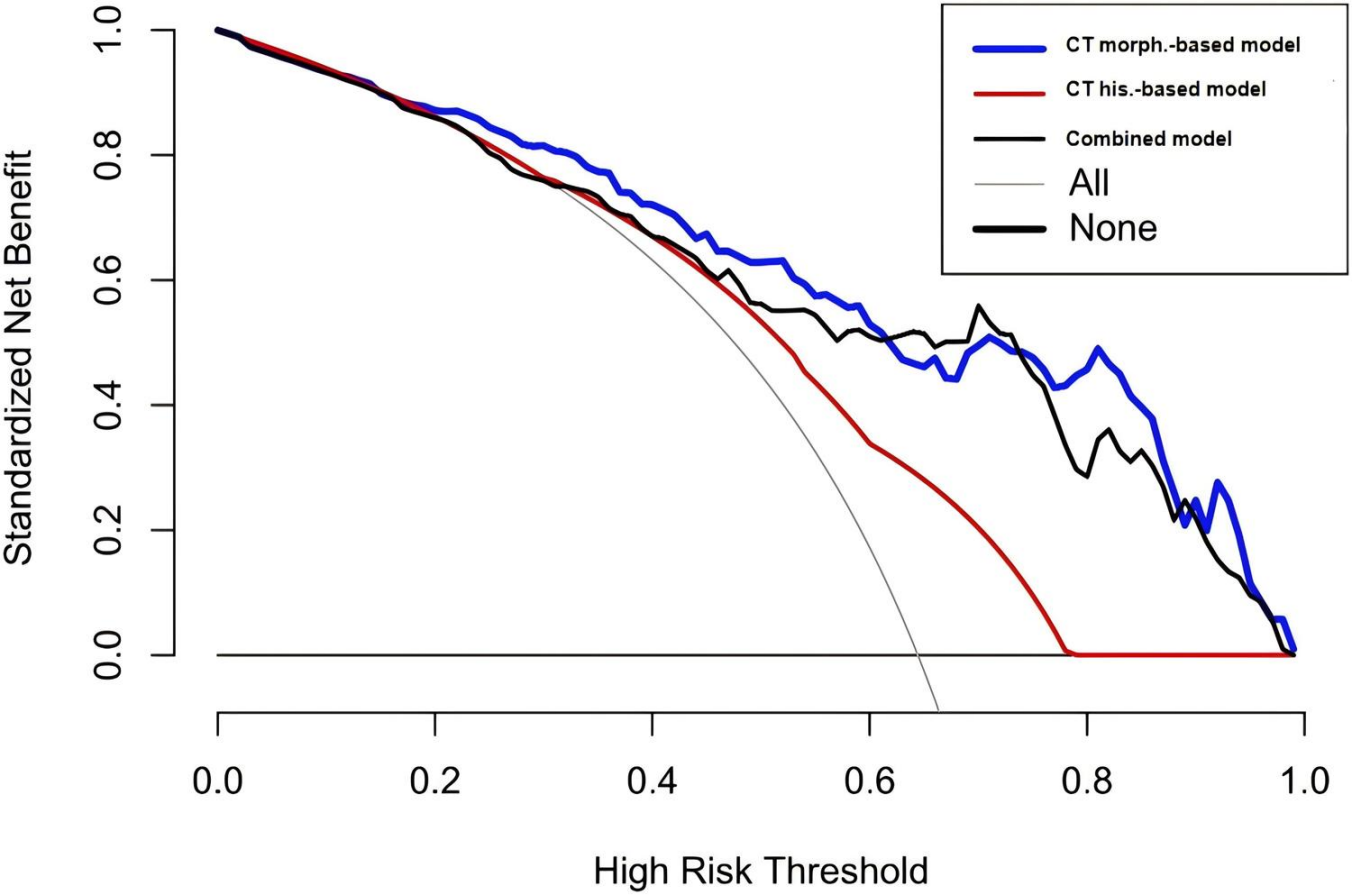
The decision curve analysis of different models. The blue line represents the combined model, the red line represents the CT morphological feature–based model, and the black line represents the CT histogram–based model. The *x*-axis indicates the threshold probability, and the *y*-axis represents the standardized net benefit. Overall, the combined model demonstrates superior clinical benefit compared with the CT morphological feature–based model and the CT histogram-based model.

The independent predictors derived from the CT feature-based model and the histogram-based model were subsequently included in a multivariable logistic regression analysis to construct the combined model. Tumor location, vacuole sign, kurtosis, and the 90th percentile were identified as independent predictors of PIMA (Table 3). CT morphological and histogram features that exhibited statistical significance were simultaneously subjected to multivariate logistic regression analysis, which identified tumor location, vacuole sign, kurtosis, and the 90th percentile as independent predictors of PIMA (Table 3). These factors were used to establish a combined prediction model. Receiver operating characteristic curve analysis showed that the combined prediction model achieved an AUC of 0.845, sensitivity of 0.828, and specificity of 0.705, indicating superior diagnostic performance compared to the CT morphological and CT histogram-based prediction models (Figure 2 and Table 4). A nomogram was constructed to visualize the combined prediction model (Figure 3). Calibration curves demonstrated good agreement between the predicted probabilities and observed outcomes (Figure 4). Decision curve analysis indicated that within a certain range of threshold probabilities, the combined model provided a greater net clinical benefit than the other two models (Figure 5).

## 4 Discussion

The present study analyzed the value of CT morphological and grayscale histogram features in differentiating between PIMA and PINMA. The results demonstrated that while both CT morphological and grayscale histogram features achieved relatively high levels of discriminatory accuracy, their combined application further improved diagnostic performance. The nomogram developed in this study may serve as a practical tool to assist radiologists in preoperative differentiation between PIMA and PINMA, thereby supporting clinical decision-making.

CT morphological features can reflect the biological characteristics of tumors to some extent. In the present study, PIMA was more frequently located in the lower lobes of both lungs, whereas PINMA predominantly occurred in the upper and middle lobes. These findings were consistent with those reported by Chen (18). Previous studies have suggested that chronic and repetitive exposure to environmental factors such as smoking and air pollution may induce goblet cell hyperplasia or metaplasia. Owing to gravitational effects, mucociliary clearance is more pronounced in the lower lobes, which may increase the likelihood of goblet cell hyperplasia or metaplasia in these regions, thereby contributing to the preferential occurrence of PIMA in the lower lobes (19). In addition, the vacuole sign was more commonly observed in PIMA than in PINMA. The underlying pathological mechanism may involve drainage of mucus from mucin pools through the adjacent bronchi. Furthermore, invasive tumor growth along the terminal airways may form a check-valve mechanism, leading to airflow limitation with preferential air trapping. Subsequent alveolar over-distension and rupture may result in the formation of air-containing spaces (20, 21). Moreover, PIMA lesions in the present study were predominantly solid. This may be attributed to the abundant intracellular mucus secreted by PIMA cells into the alveolar spaces, which subsequently fills the alveolar lumen, resulting in solid attenuation on CT (22). PINMA produces relatively less mucus. When tumor cells exhibit lepidic growth or fail to completely fill alveolar spaces, residual air within the alveoli leads to the appearance of ground-glass opacity on CT.

CT grayscale histogram analysis quantifies gray-level image information and reflects tumor heterogeneity and density distribution characteristics. The results of the present study indicate that the histogram features of PIMA and PINMA can capture the underlying histological distinctions. Specifically, the histogram characteristics of PIMA, such as higher mean, maximum, and uniformity values than those of PINMA, suggest higher attenuation and a more homogeneous density distribution. This may be related to the pathological features of PIMA, which is predominantly composed of goblet and/or columnar tumor cells with abundant intracellular mucin, while cellular atypia is typically mild or absent (23). The higher entropy and skewness values exhibited by PINMA compared to PIMA reflect greater density variability and an asymmetric gray-level distribution. This may be attributable to the marked histological heterogeneity of PINMA, which is characterized by complex combinations of architectural growth patterns in varying proportions (24). No significant differences were observed between the two groups with respect to certain parameters, such as the minimum value. This may be partly explained by the technical limitations of ROI delineation, particularly the incomplete exclusion of adjacent normal lung tissue. Further optimization of lesion segmentation methods may help reduce measurement bias in future studies.

Although radiomics has been widely used in lung cancer (25–27), CT histogram analysis offers higher clinical feasibility. Radiomics requires complex data processing and algorithmic support, whereas histogram analysis, which relies on gray-scale statistical parameters, is easier to implement and more suitable for routine clinical practice. CT histogram analysis focuses on the quantitative description of the tumor microenvironment, which can compensate for the shortcomings of the simple morphological analysis performed in conventional CT. Future studies may incorporate additional quantitative imaging features, such as radiomics or deep learning-based features, to further improve diagnostic performance. The combined prediction model established in this study using CT morphological and histogram-based features distinguished PIMA from PINMA with good diagnostic accuracy. In addition, the nomogram constructed to visualize the combined prediction model provides an intuitive and non-invasive assessment tool for the clinical differentiation of PIMA and PINMA and is particularly suitable for patients without surgical indications. Although the model demonstrated promising diagnostic performance, external validation using independent datasets is still required to further confirm its robustness and clinical applicability.

This study had several limitations. First, its single-center, retrospective design may have introduced selection bias. Second, the sample size was limited by the relative rarity of PIMA, which may have affected the statistical robustness and generalizability of the of the results. Third, internal validation, such as bootstrapping or cross-validation, was not performed, which may limit the assessment of model stability and potential optimism. Fourth, the subjective nature of the ROI delineation may have introduced observer-related bias. Future multicenter studies with larger cohorts and more precise automated segmentation algorithms are warranted to further validate our findings and minimize the influence of human factors. In addition, automated or semi-automated segmentation techniques should be incorporated in future studies to reduce human-related variability and improve reproducibility.

## 5 Conclusion

In conclusion, this study demonstrates the potential value of using CT morphological and histogram features to differentiate between PIMA and PINMA, with their combined application further enhancing diagnostic performance. Visualization of the combined prediction model in the form of a nomogram provides a convenient tool for clinical practice, which may facilitate the development of non-invasive diagnostic approaches and offer meaningful support for individualized treatment strategies in patients with lung adenocarcinoma.

## Data Availability

The original contributions presented in the study are included in the article/Supplementary Material, further inquiries can be directed to the corresponding author.
